# The larger patellar tilt angle and lower intercondylar notch angle might increase posterior cruciate ligament injury risk: a retrospective comparative study

**DOI:** 10.1186/s12891-023-07054-w

**Published:** 2023-12-01

**Authors:** Zhenhui Huo, Kuo Hao, Chongyi Fan, Kehan Li, Ming Li, Fei Wang, Yingzhen Niu

**Affiliations:** Department of Orthopaedic Surgery, Third Hospital of Hebei Medical University, 139 Ziqiang Road, Shijiazhuang, 050051 Hebei People’s Republic of China

**Keywords:** Posterior cruciate ligament, Morphological risk factors, Femoral condyle, Patellofemoral alignment, Intercondylar notch, Patellar tilt angle

## Abstract

**Background:**

Posterior cruciate ligament (PCL) injuries are common ligament injuries of the knee, and previous studies often focused on the associations between the morphology of the knee and PCL injuries. Studies on the correlation between PCL injuries and patellofemoral alignment are limited.

**Methods:**

This retrospective study included 92 patients with PCL injured and 92 patients with PCL intact. Measurement parameters were compared between the two groups, including patellar tilt angle, congruence angle, patellar height, hip-knee-ankle angle, lateral trochlear inclination, femoral condyle ratio, bicondylar width, intercondylar notch width and index, notch angle, trochlear facet asymmetry, and trochlear sulcus depth and angle. Independent risk factors associated with PCL injuries were identified by logistic regression analyses.

**Results:**

In the PCL injured group, the patellar tilt angle was significantly larger (13.19 ± 5.90° vs. 10.02 ± 4.95°, *P* = 0.04); the intercondylar notch angle was significantly lower (60.97 ± 7.83° vs. 67.01 ± 6.00°, *P* = 0.004); the medial and lateral femoral condyle ratio were significantly larger (0.63 ± 0.64 vs. 0.60 ± 0.56, *P* = 0.031; 0.65 ± 0.60 vs. 0.58 ± 0.53, *P* = 0.005) than in the PCL intact group. There were 11 patients with patellar dislocation in the PCL injured group, accounting for 12%. In these patients, the patellar height was higher (1.39 ± 0.17 vs. 1.09 ± 0.25, *P* = 0.009); the trochlear sulcus angle was larger (157.70 ± 8.7° vs. 141.80 ± 8.78°, *P* < 0.001); and the trochlear sulcus depth was shallower (3.10 ± 1.20mm vs. 5.11 ± 1.48mm, *P* = 0.003) than those in the patients without patellar dislocation. Multivariate analyses showed that patellar tilt angle (each increase 1 degree, OR = 1.14) and intercondylar notch angle (each increase 1 degree, OR = 0.90) were independent risk factors for PCL injuries.

**Conclusion:**

The patients with PCL injuries had larger patellar tilt angles, lower intercondylar notch angles, and longer posterior femoral condyles than patients with PCL intact. The larger patellar tilt angle and lower intercondylar notch angle might be risk factors for PCL injuries.

## Background

Posterior cruciate ligament (PCL) injuries account for approximately 1–40% of all sports-related traumatic knee injuries, depending on the type of sport [1, 2]. With the growing number of athletes and especially of competitive athletes, the absolute number of PCL injuries is also growing and may continue to grow in the next few years [3–5]. In most cases, PCL rupture causes pain and posterior laxity and decreases a person’s ability to participate in sports in the short term. In the long term, the absence of PCL will lead to abnormal kinematics and increased contact pressures in the medial and patellofemoral compartments of the knee and may increase strain on the posterolateral knee structures, placing them at risk of subsequent injury [6, 7]. The posterolateral corner structure, which shows the greatest increase in force with deficiency of PCL under both gait- and squat-loading conditions, is the popliteus tendon [6]. Injuries to the posterolateral corner structure require surgical repair and / or reconstruction [8, 9]. Many current knee morphologic studies have focused on anterior cruciate ligament (ACL) injuries. Although most risk factors are difficult to eliminate during surgery, identifying risk factors is critical in preventing injuries and re-injuries after ligament reconstruction. A meta-analysis presented by Yulun et al. [10] reported that ACL injury prevention programs can significantly reduce injury rates, and developing prevention programs requires a thorough understanding of risk factors, which is a prerequisite.

Even though we understand some mechanisms behind PCL injuries, morphological risk factors for PCL injuries remain unclear. We study the morphological risk factors of PCL injuries because it is helpful to screen patients (such as athletes) prone to PCL rupture. An improved understanding of these factors enhances screening for individuals prone to PCL injury [11]. For these susceptible people, we can develop personalized training programs and exercise methods, as specific training programs have been used to prevent ACL injuries in athletes [12–14]. Patients who are at higher risk have the opportunity to receive counseling, supplementary education, early intervention, and therapeutic measures.

There remains ongoing debate surrounding the risk factors associated with PCL injuries within the academic community [15, 16]. In recent years, based on advances in biomechanical and morphological studies, a decrease in the posterior tibial slope, a smaller and more sharply angled intercondylar notch, and a smaller notch width index (NWI) are considered to be the knee morphological risk factors for PCL injuries [11, 17, 18]. One of the controversial risk factors for PCL injuries is the intercondylar notch. Van Kuijk et al. [17] first examined the morphological characteristics of patients with PCL rupture. They used statistical shape modeling software and reported that a sharper intercondylar notch and flatter tibial eminence are risk factors for PCL rupture. Huang et al. [15] noted that decreased notch width index was associated with an increased incidence of PCL injured. Fan et al. [18] reported that coronal NWI is related to PCL avulsion fracture in the female population. Liu et al. [16] concluded that a larger coronal notch width index was the greatest risk factor for posterior cruciate ligament rupture in the female population. The results did not indicate that patients with a PCL rupture have a stenotic intercondylar notch. Although contact injuries account for a high proportion of PCL injuries [19, 20] and PCL injuries are often directly caused by high violence, these findings still emphasize the importance of knee anatomical morphology for PCL injuries.

The intercondylar notch as an integral part of the femoral condyle has an effect on the PCL. The femoral condyle is involved in the patellofemoral alignment. Whether the abnormal patellofemoral malalignment has an effect on the PCL remains unknown. Hao et al. [21] reported a direct correlation between patellofemoral malalignment and narrowed intercondylar notch. They concluded that patients with patellar instability had a stenotic intercondylar notch and a thin ACL. Patellar instability is often associated with patellofemoral malalignment, patellar displacement, and stenotic intercondylar notch. However, the relationship between PCL injuries and patellofemoral alignment is still worthy of attention. Currently, a limited number of research teams are dedicated to conducting specialized investigations, and the relationship between the two still needs to be clarified.

The main purpose of this study was to investigate the relationship between the patellofemoral alignment and the morphology of the femoral condyle and PCL injuries. In this way, we can summarize the risk factors that may lead to PCL injury, provide early warning for clinicians, respond in advance, and provide tailored treatment options for patients. We assume that people with a larger patellar tilt angle and lower intercondylar notch angle are more likely to have PCL injuries.

## Materials and methods

### Patient selection

**Fig. 1:**
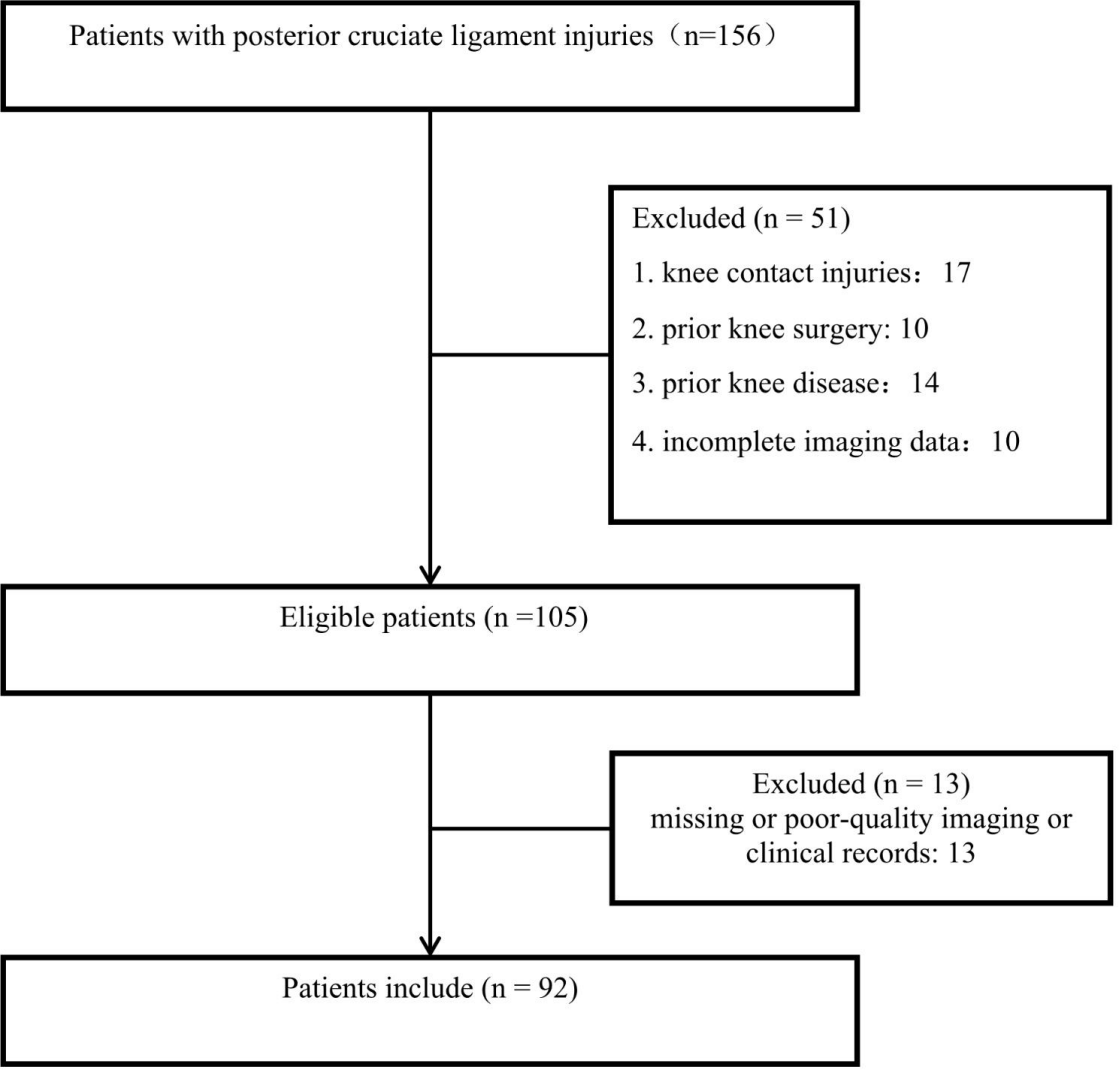
Flowchart of patient inclusion and exclusion in the PCL injured group

This study retrospectively collected the imaging data of patients with knee diseases admitted to the Third Hospital of Hebei Medical University from November 2022 to March 2023. This retrospective study was approved by the Ethics Committee of our hospital and obtained written informed consent from all included patients. The PCL injured group was enrolled in the study based on the presence of the following criteria: (1) 18–45 years old; (2) Knee joint injuries or discomfort caused by non-contact mechanisms such as manual labor and sudden stop and swerve in sports activities; (3) PCL injuries confirmed by arthroscopic exploration or magnetic resonance imaging (MRI); (4) Patients with knee osteoarthritis Kellgren-Lawrence grade ≤ 1 [22]. Exclusion criteria : (1) Knee injuries caused by contact mechanisms such as falling from height and vehicle collision; (2) Patients with incomplete PCL tear confirmed by radiographs and MRI examination and arthroscopic surgery; (3) Patients with knee diseases such as knee joint tuberculosis, suppurative arthritis, rheumatism or rheumatoid arthritis; (4) Patients who had a history of knee trauma and knee surgery such as distal femoral fracture, tibial plateau fracture or open knee trauma; (5) Patients with incomplete MRI and radiographs films. The flow chart of patient selection in the PCL injured group is shown in Fig. 1.

The PCL intact group was screened from the case system of our hospital and selected from patients without PCL rupture in a 1:1 ratio matched by age, sex, height, and weight during the same period for comparison. The PCL intact group was patients with no apparent PCL injuries found by physical examination, radiographs, and MRI examination. Patients with ligament laxity, meniscus injuries, patellar trajectory abnormalities and abnormal anatomical morphology of knee joints, such as a tumor or discoid meniscus, were excluded. Patients with knee osteoarthritis Kellgren-Lawrence grade > 1, previous history of knee joint trauma, and knee joint surgery were also excluded.

According to the inclusion and exclusion criteria, 92 patients (92 knees) were included in the PCL injured group, and 92 patients (92 knees) were included in the PCL intact group. Demographic data of all patients were collected, including age, gender, height, weight, side, and time from injuries to imaging.

### Computed tomography (CT) and radiography

All CT examinations were performed using a multidetector CT scanner (Lightspeed VCT 64, GE, Milwaukee, WI, USA) with a 0.625-mm reconstructive slice and interval thickness. Participants were examined in the supine position with their knees extended. All study participants underwent full weightbearing long-leg standing and lateral knee radiographs according to a standardized protocol to avoid bias [23].

### Measurements

All CT scans and radiographs were reviewed and evaluated carefully by two skilled and independent researchers using RadiAnt-DICOM software (Medixant Ltd., Poznan, Poland), which has an accuracy of 0.01 mm for distance and 0.01° for angles. All of the images were evaluated in a random order. Two researchers were unaware of the patient information, grouping, study purpose, and hypothesis. The average measurements were used for final analyses to minimize the measurement error. Any disagreement was resolved through a discussion with another senior researcher until the consensus was reached. Measurements were performed using a standardized technique described in detail below. The reliability of all measurements was evaluated using intraclass correlation coefficient (ICC) values with 95% confidence intervals. All measurements were performed by two independent researchers to ensure interobserver reliability. To assess intraobserver reliability, one researcher repeated all measurements with an interval of three weeks. The ICC values ≥ 0.8 were considered as good, ≥ 0.9 were considered as excellent [18].

#### Patellofemoral alignment

**Fig. 2:**
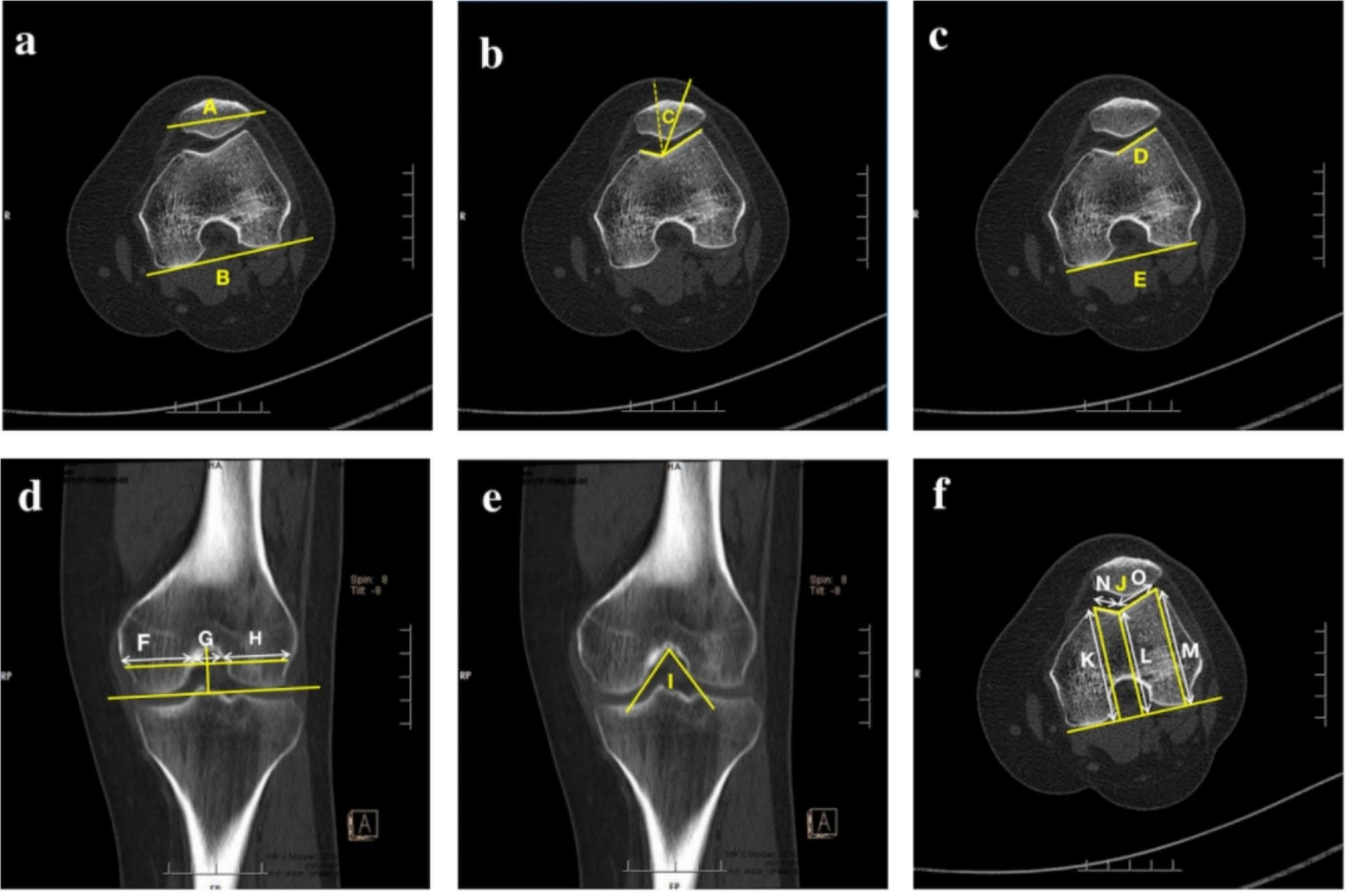
Measurements of patellofemoral alignment in the a-b planes and morphology of the femoral condyle in the c-f planes. **a**. The patellar tilt angle was the angle between line **A** which was drawn along the longest axis of the patella and the femoral posterior condylar line **B**. **b**. The congruence angle **C** was the angle of the bisector of the trochlear groove angle and the dotted line. **c**. The lateral trochlear inclination was the angle between the posterior condylar line **E** and line **D** along the lateral facets. **d**. A posterior condylar line which connected the posterior margins of the medial and lateral femoral condyles was determined. All femoral widths are measured parallel to this reference line. The intercondylar notch height was the distance from the top of the intercondylar notch to the posterior condylar line. The intercondylar notch width **G** was measured at the anterior third of the notch height. At the same level, the medial condylar width **F** and lateral condylar width **H** were measured. The bicondylar width was the sum of the medial condylar width **F**, notch width **G**, and lateral condylar width **H**. The NWI was the ratio of the notch width **G** to the bicondylar width. **e**. The notch angle **I** was formed between the two tangential lines of the entrance of the medial and lateral femoral condyles from the top of the intercondylar notch. **f**. The trochlear sulcus angle **J** was the angle contained by the medial and lateral trochlear facets interacting at the deepest point. The trochlear sulcus depth was the difference between the mean value of the distance **K**, **M** from the highest point of the medial and lateral trochlear surface to the tangent of the femoral posterior condylar and the distance **L** from the trochlear groove to the tangent of the posterior condylar. The trochlear facet asymmetry was the ratio of the lateral trochlear width **O** and medial trochlear width **N**. NWI, notch width index

(1) Patellar tilt angle: Using an axial CT scan of the knee, when the intercondylar.

shows the level of the “Roman arch”, a posterior condylar line that connects the posterior margins of the medial and lateral femoral condyles was determined. A second line is drawn along the longest axis of the patella. The angle of the two lines on the transverse axis is defined as the patellar tilt angle (Fig. 2a).

**Fig. 3:**
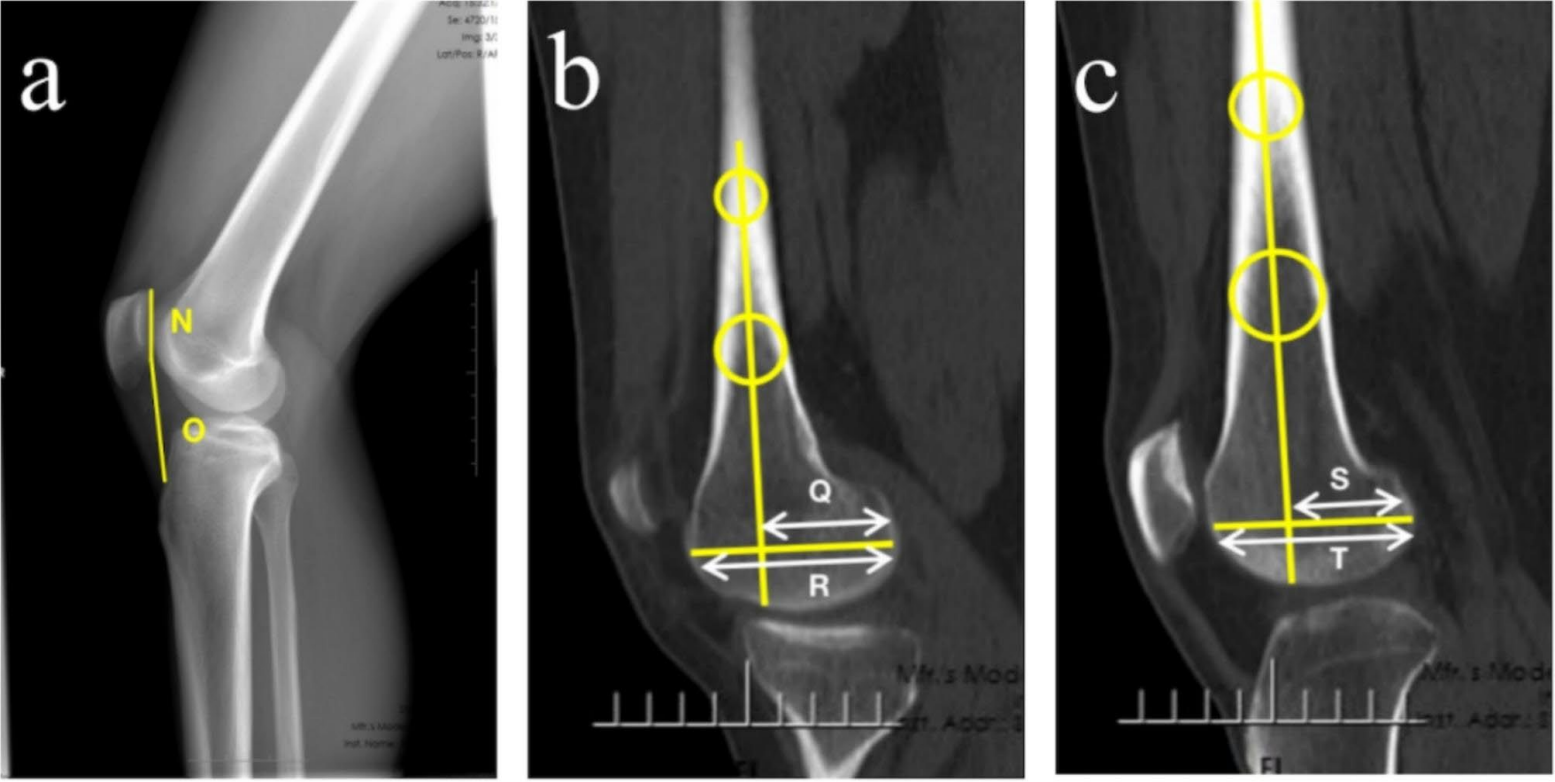
Measurements of patellar height in the **a** plane and femoral condyle ratio in the **b-c** planes. **a**. The patellar height (Caton–Deschamps index) was the ratio of the length **N** from the distal edge of the patella articular surface to the anterosuperior angle of the tibial plateau to the length **O** of the patella articular surface. **b**, **c**. The medial or lateral femoral condyle ratio was defined as the ratio of the distance **Q**, **S** from the intersection of the two axes to the most posterior point of the medial or lateral condyle, to the total anteroposterior distance **R**, **T** of the medial or lateral condyle

**Fig. 4:**
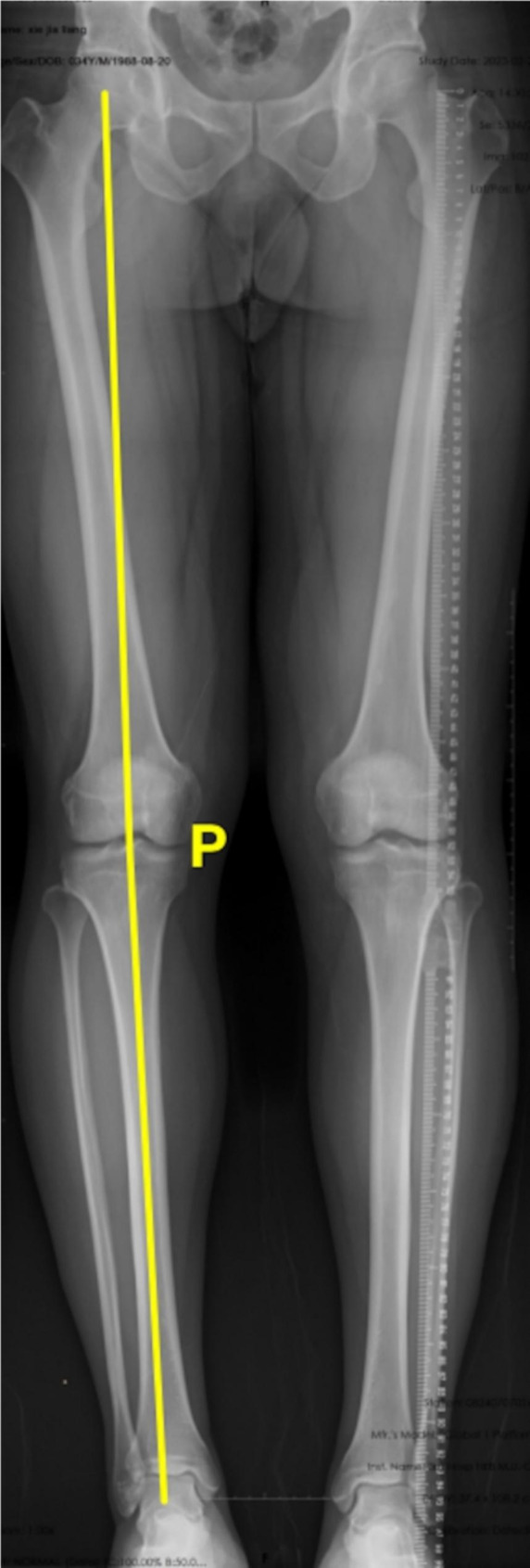
Measurements of HKA. HKA was the angle **P** between the line from the center of the hip joint to the center of the knee joint and the line from the center of the knee joint to the center of the ankle joint. HKA, Hip–knee–ankle angle

(2) Congruence angle: The congruence angle is measured by using axial CT scans of the knee. A line is drawn through the lower pole of the patella and the deepest point of the trochlear groove. The congruence angle is the angle between the line and the bisector of the trochlear groove angle. This angle is negative on the medial side of the bisector and positive on the lateral side [24] (Fig. 2b).

(3) Patellar height (Caton-Deschamps index): Caton-Deschamps index is the ratio of the length from the distal edge of the patella articular surface to the anterosuperior angle of the tibial plateau to the length of the patella articular surface on the lateral radiographs of the knee at flexion of 30 ° (Fig. 3a).

(4) Hip–knee–ankle angle (HKA): HKA is defined as the angle between the line from the center of the hip joint to the center of the knee joint and the line from the center of the knee joint to the center of the ankle joint [25] (Fig. 4).

#### Morphology of the femoral condyle

(1) Lateral trochlear inclination: Lateral trochlear inclination is the angle between the posterior condylar line and the line along the lateral facets [26] (Fig. 2c).

(2) Intercondylar notch width, bicondylar width, NWI, bicondylar width, and intercondylar notch angle: On the coronal view, the slice with the highest tibial spine is chosen for measurements. A posterior condylar line which connects the posterior margins of the medial and lateral femoral condyles is determined. All femoral widths are measured parallel to this reference line. The intercondylar notch height is defined as the distance between the top of the intercondylar notch and the posterior condylar line. The notch width is measured at the anterior third of the notch height, which is defined as the distance between the medial and lateral edges of the intercondylar notch. The bicondylar width is the sum of the medial condylar width, notch width, and lateral condylar width. NWI is the ratio of the notch width to the bicondylar width. The intercondylar notch angle is defined as the angle between the two tangential lines of the entrance of the medial and lateral femoral condyles from the top of the intercondylar notch [21] (Fig. 2d and e).

(3) Trochlear sulcus depth, sulcus angle, and trochlear facet asymmetry: The trochlear sulcus angle is the angle contained by the medial and lateral trochlear facets interacting at the deepest point. Sulcus depth is the difference between the mean value of the distance from the highest point of the medial and lateral trochlear surface to the tangent of the femoral posterior condylar and the distance from the trochlear groove to the tangent of the posterior condyle. The trochlear facet asymmetry is the ratio of the lateral trochlear width and medial trochlear width [27] (Fig. 2f).

(4) Medial or lateral femoral condyle ratio (MFCR/LFCR): Two separate circles with a distance of 5 cm are determined in the center of the femoral shaft. The more distal one is positioned at the most proximal aspect of the trochlear. The line passing through the centers of the two circles is determined to identify the long axis of the distal femur. The line between the most posterior point and the most anterior point of the femoral condyle is determined to identify the axis of the medial or lateral femoral condyle. The medial or lateral femoral condyle ratio is defined as the ratio of the distance from the intersection of the two axes to the most posterior point of the medial or lateral condyle to the total anteroposterior distance of the medial or lateral condyle [28] (Fig. 3b and c).

### Statistical analysis

Measurement data were expressed as the means ± standard deviations for continuous variables, and counts for categorical variables. The Levene’s test and Kolmogorov-Smirnov test were used to check the homogeneity and normality of the data. All numerical variables showed a normal distribution and equal variance. The independent samples t-test was performed to examine differences in continuous variables (patellar tilt angle, congruence angle, patellar height, HKA, lateral trochlear inclination, medial and lateral femoral condyle ratio, bicondylar width, intercondylar notch width, NWI, notch angle, trochlear facet asymmetry, trochlear sulcus depth, and trochlear sulcus angle). The data were analyzed using IBM SPSS version 26.0 (Statistical Package for the Social Sciences, Inc.; Chicago, IL). Differences were considered significant at *P* < 0.05.

The parameters with significant differences between the two groups were included in the multivariate logistic regression analysis. *P* < 0.05 was considered statistically significant.

The minimum sample size was calculated with an a priori power calculation using G*Power version 3.1.9.4 (Heinrich-Heine-Universitat Dusseldorf, Dusseldorf, Germany). To achieve a power of 80% and a two-sided significance level of 0.05, a minimum of 34 samples per group was needed to detect a 1° angle difference.

## Results

A total of 156 patients with PCL injuries were treated during the study period. 10 patients had previously undergone knee surgery, 14 had a history of knee osteoarthritis Kellgren-Lawrence grade > 1, rheumatoid arthritis, and other disorders, and the imaging data for ten patients was insufficient. Moreover, 17 cases of PCL injuries were caused by the contact injury mechanism (Fig. 1).

The study involved a total of 184 patients (184 knees): 92 patients (92 knees) in the PCL injured group, including 52 males and 40 females, with an average age of 25.03 ± 5.70 years; there were 92 patients (92 knees) in the PCL intact group, including 52 males and 40 females, with the average age of 25.92 ± 6.20 years. Between the two groups, there was no significant difference regarding demographic information between the PCL injured group and PCL intact group (*P* > 0.05) (Table 1).

**Table 1:** Baseline clinical characteristics between the two study groups

Variable	PCL injured (n = 92)	PCL intact (n = 92)	*P* value
Number of knees, n	92	92	-
Number of patients, n	92	92	-
Sex (male/female), n	52/40	52/40	-
Side (left: right), n	42/50	51/41	0.744
Age, y	25.03 ± 5.70	25.92 ± 6.20	0.603
BMI, kg/m^2^	26.27 ± 3.48	25.80 ± 5.08	0.291
MFITI, d	10.71 ± 16	11.94 ± 10	0.340

Data are presented as the mean ± standard deviation

Bold indicates statistical significance (*P* < 0.05)

PCL, Posterior cruciate ligament; BMI, Body mass index; MFITI, Time from injuries to imaging

The ICC values for all measured parameters were good to excellent, with intra-observer ICC values ranging from 0.823 to 0.912 and inter-observer ICC values ranging from 0.819 to 0.947, indicating strong intra- and inter-observer reliability for all measurements.

The patellar tilt angle of the PCL injured group was significantly larger than that of the PCL intact group (13.19 ± 5.90° vs. 10.02 ± 4.95°, *P* = 0.04). In comparison to the intact group, the intercondylar notch angle of the PCL injured group was noticeably lower (60.97 ± 7.83° vs. 67.01 ± 6.00°, *P* = 0.004). The femoral condyle ratio in PCL injured group was significantly larger than that in PCL intact group (medial condyle length ratio: 0.63 ± 0.64 vs. 0.60 ± 0.56, *P* = 0.031; lateral condyle length ratio: 0.65 ± 0.60 vs. 0.58 ± 0.53, *P* = 0.005). Among them, there were no statistical differences in congruence angle, patellar height, HKA, lateral trochlear inclination, bicondylar width, intercondylar notch width, NWI, trochlear facet asymmetry, trochlear sulcus depth, and trochlear sulcus angle between the PCL injured group and the PCL intact group (*P* > 0.05) (Table 2).

**Table 2:** Comparison of the patellofemoral alignment and femoral condyle morphological parameters between the two study groups

Variable	PCL injured (n = 92)	PCL intact (n = 92)	*P* value
Patellar lateral tilt, deg	13.19 ± 5.90	10.02 ± 4.95	**0.043**
Congruence angle, deg	-12.36 ± 14.21	-13.25 ± 19.61	0.836
Patellar height, CDI	1.14 ± 0.19	1.04 ± 0.21	0.073
Hip–knee–ankle angle, deg	179.72 ± 2.75	179.37 ± 2.56	0.659
Lateral trochlear inclination, deg	19.42 ± 5.27	19.08 ± 4.74	0.802
Trochlear facet asymmetry	0.62 ± 0.15	0.62 ± 0.14	0.993
Intercondylar notch width, mm	20.31 ± 2.29	20.20 ± 2.21	0.846
Bicondylar width, mm	75.99 ± 6.20	72.92 ± 6.85	0.078
Notch width index	0.27 ± 0.03	0.28 ± 0.02	0.222
Intercondylar notch angle, deg	60.97 ± 7.83	67.01 ± 6.00	**0.004**
Trochlear sulcus angle, deg	143.70 ± 9.78	146.34 ± 10.59	0.33
Trochlear sulcus depth, mm	4.87 ± 1.86	4.16 ± 1.41	0.134
Lateral femoral condyle ratio	0.65 ± 0.60	0.58 ± 0.53	**0.005**
Medial femoral condyle ratio	0.63 ± 0.64	0.60 ± 0.56	**0.031**

Data are presented as the mean ± standard deviation

Bold indicates statistical significance (*P* < 0.05)

CDI, Caton-Deschamps Index

Eleven patients with patellar dislocation were observed, accounting for 12% of the PCL injured group. A subgroup comparison was conducted in the PCL injured group to examine the differences between patients with PCL injuries with or without patellar dislocation. The parameters of the comparison between the two groups shown in Table 3. It was found that patients with patellar dislocation had a higher patellar position compared to patients without patellar dislocation (1.39 ± 0.17 vs. 1.09 ± 0.25, *P* = 0.009). Additionally, the trochlear sulcus angle was larger in patients with patellar dislocation than those without patellar dislocation (157.70 ± 8.7° vs. 141.80 ± 8.78°, *P* < 0.001). Furthermore, the trochlear sulcus depth was shallower in patients with patellar dislocation compared to those without patellar dislocation (3.10 ± 1.20mm vs. 5.11 ± 1.48mm, *P* = 0.003). In contrast, there were no statistically significant differences in patellar tilt angle, congruence angle, HKA, lateral trochlear inclination, medial and lateral femoral condyle ratio, bicondylar width, intercondylar notch width, NWI, notch angle, trochlear facet asymmetry (*P* > 0.05).

**Table 3:** Multifactorial logistic regression analysis of risk factors associated with PCL injuries

Variable	OR	95%CI	*P* value
Patellar lateral tilt, deg	1.14	1.00 to 1.29	**0.040**
Intercondylar notch angle, deg	0.90	0.82 to 0.98	**0.014**
Lateral femoral condyle ratio	4.79	4.85 to 5.35	0.230
Medial femoral condyle ratio	4.00	3.11 to 4.68	0.905

Bold indicates statistical significance (*P* < 0.05)

Multivariate logistic regression analysis was used to verify the relationship between patellar tilt angle, femoral condyle ratio, intercondylar notch angle and PCL injuries. In the multivariate logistic regression model analysis, the larger patellar tilt angle (each increase 1 degree, OR = 1.14) and lower intercondylar notch angle (each increase 1 degree, OR = 0.90) were independent risk factors for PCL injuries (Table 4).

**Table 4:** Comparisons of the measurement parameters between the PCL injured groups with and without PD

Variable	PCL injured with PD (n = 11)	PCL injured without PD (n = 81)	*P* value
Patellar lateral tilt, deg	13.56 ± 5.70	13.14 ± 4.97	0.089
Congruence angle, deg	-8.54 ± 6.21	-12.72 ± 5.61	0.078
Patellar height, CDI	1.39 ± 0.17	1.09 ± 0.25	**0.009**
Hip–knee–ankle angle, deg	179.87 ± 2.76	179.70 ± 3.00	0.659
Lateral trochlear inclination, deg	11.54 ± 5.41	20.49 ± 4.15	0.245
Trochlear facet asymmetry	0.40 ± 0.14	0.65 ± 0.13	0.736
Intercondylar notch width, mm	19.35 ± 3.19	20.44 ± 2.87	0.465
Bicondylar width, mm	74.37 ± 6.27	76.21 ± 6.65	0.134
Notch width index	0.27 ± 0.02	0.27 ± 0.02	0.086
Intercondylar notch angle, deg	61.93 ± 7.39	60.84 ± 6.89	0.055
Trochlear sulcus angle, deg	157.70 ± 8.77	141.80 ± 8.78	**< 0.001**
Trochlear sulcus depth, mm	3.10 ± 1.20	5.11 ± 1.48	**0.003**
Lateral femoral condyle ratio	0.63 ± 0.53	0.65 ± 0.63	0.658
Medial femoral condyle ratio	0.64 ± 0.43	0.63 ± 0.61	0.320

Data are presented as the mean ± standard deviation

Bold indicates statistical significance (*P* < 0.05)

PD, patellar dislocation; CDI, Caton-Deschamps Index

## Discussion

The most important finding of the present study was that patients with PCL injuries had a larger patellar tilt angle, a lower intercondylar notch angle, and a larger femoral condyle ratio than those with PCL integrity. This was the first study to link patellofemoral alignment to PCL injuries.

Previous studies have shown that PCL injuries might be related to smaller and sharper intercondylar notch, smaller intercondylar notch width, smaller intercondylar notch volume, and smaller posterior tibial slope [17, 29–31]. Van Kuijk et al. [17] first examined the morphological characteristics of patients with PCL rupture. They believed a smaller intercondylar width with a sharper angle was associated with PCL rupture. Our study found that lower intercondylar notch angle was associated with PCL injury, which was consistent with his findings. In another study [29], they found that there were no significant differences in the bicondylar width, notch width, and notch width index between patients with a ruptured PCL and control patients. Our study also found that there was no significant difference in these three morphological indicators between the two groups, which was the same as their result.

The intercondylar notch has been a topic of interest. A sharper intercondylar notch angle is often considered a risk factor for ACL injuries. The smaller the intercondylar notch height and the narrower the intercondylar notch width, the smaller the intercondylar volume and the greater the possible risk of PCL injuries. Our study found that the intercondylar notch angle of the PCL injured group was lower than that of the PCL intact group. The size of the PCL (as well as the ACL) is associated with the size of the intercondylar notch [32, 33]. It is well accepted that the volumes of the intercondylar notch, the PCL, and the ACL are all positively correlated. When the PCL is smaller, it has a lower capacity to withstand force and is more susceptible to tearing [34, 35]. An alternative interpretation is that a reduced intercondylar notch size might lead to impingement on the PCL. Triantafyllidi et al. [36] demonstrated that the PCL occupied a significant portion of the intercondylar notch during flexion, potentially resulting in impingement on the PCL in this joint position. However, it is still unclear whether the PCL injuries are due to the relatively weak tensile strength possessed by the smaller volume of the ligament or to chronic or acute injuries caused by intercondylar notch impingement of the ligament [37–40]. Due to the anatomical position of the PCL behind the knee joint, the impact of the incision may have less effect on the PCL rupture. However, biomechanical studies are required to clarify the mechanism of PCL injuries further. Liu et al. [16] believed a larger intercondylar notch width index was the most significant risk factor for PCL rupture in the female population. The reasons for his opposite conclusions may be as follows: different from the control group in Liu’s study, people with trauma but no knee joint structural injuries may have relatively thick ligaments and wide intercondylar notch; their study included cases of partial PCL injuries. These are all reasons that may differ from our study.

Previous studies have shown that ACL injuries are associated with an increased ratio of the distal lateral femoral condyle [27, 41, 42]. In the study of Hao et al. [21], it was found that the LFCR of posterior femoral condyle depth was significantly larger in patellar instability patients, which was a risk factor for ACL injury. In our research, it was observed that an augmented depth of both the medial and lateral posterior femoral condyles exhibited a correlation with PCL injuries. It might be related to the influence of distal femur morphology on knee joint kinematics [30, 43]. Altered tibiofemoral interaction due to increased posterior femoral condyle depth may lead to altered gait and loading mechanics [44]. This may increase the risk of PCL injuries. However, there are no relevant studies to demonstrate the mechanism of action of the increased length of the medial posterior femoral condyle on PCL injuries, which is a shortcoming of our article. Our study found that the larger patellar tilt angle was closely related to PCL injuries. Similar conclusions are rarely found in previous studies and are not even mentioned in the study of ACL injuries. The patellar tilt angle is often a good indicator for predicting patellar instability [45]. However, the biomechanical mechanism of PCL injuries caused by increased patellar tilt still requires to be further studied.

In our case, we found 11 patients with patellar dislocation. By comparing the two groups of patients with PCL injuries with or without patellar dislocation, we found that in patients with patellar dislocation, the patellar position was higher, the trochlear groove angle was larger than in patients with PCL injuries without patellar dislocation, and the trochlear depth was shallower than in patients with PCL injuries without patellar dislocation. Askenberger et al. [46] conducted a prospective study to evaluate the patellofemoral joint morphology of 103 cases of patellar dislocation and 69 cases of skeletal immature children without patellar dislocation for 2.5 years. The results showed significant differences in the two groups’ measurement parameters related to trochlear dysplasia. Among them, the patients with patellar dislocation had a larger trochlear groove angle and lower trochlear depth (< 3 mm), and the patellar height was significantly higher than those in the control group, which was consistent with our findings.

In recent years, after studying the relationship between anterior knee pain and patellofemoral alignment, many scholars have found that abnormal patellofemoral alignment (patellar subluxation and patellar tilt) caused patellofemoral disorder, resulting in articular cartilage damage of the patella and eventually leading to advanced osteoarthritis [47, 48]. Studies found that PCL rupture can lead to abnormal movement and increased contact pressure in the medial compartment of the knee joint and the patellofemoral joint, accelerating its degenerative changes and devastating long-term impacts on the knee joint [26, 49]. Our study discussed the relationship between PCL rupture and patellofemoral malalignment using the measurement techniques established in the existing literature.

A meta-analysis by Yulun et al. [10] reported that ACL injury prevention programs can significantly reduce injury rates. There appeared to be no prevention programs for PCL injury, but only as basic research progresses would these programs emerge [29]. The premise of formulating prevention programs is to determine risk factors. After confirming the influencing factors of patellofemoral malalignment and anatomical morphology of femoral condyle on PCL injuries and re-injuries after PCL graft healing and reconstruction through imaging screening, personalized prevention programs are formulated for high-risk groups. Additionally, tailored surgical plans and rehabilitation strategies can be devised for patients to rectify abnormal anatomical morphology. Through the results of this study, clinicians can comprehensively consider the risks of PCL injuries by measuring patellar tilt angle, intercondylar notch angle, and femoral condyle ratio through imaging data to evaluate the risk population of PCL injuries and give preventive suggestions and guidance for the treatment. We are convinced that this study is an important step in identifying risk factors, and we hope that we have inspired researchers to investigate the results we found further.

There are some limitations to this study. First, the trial is a retrospective case-control study with a relatively low level of evidence. Second, the study only evaluated patients with PCL lesions in Hebei Province, China. Studies that take the variability across regions and ethnicities into account were required. Third, it is difficult to achieve the complete consistency of the direction, size, and mechanism of the force when the injuries occurred. There are still confounding factors that affected the study’s results, such as the site of non-contact injuries, the shoes worn, and other factors that were not controlled. There is no good distinction between the four mechanisms of PCL injuries: dashboard injuries, severe torsion with valgus or varus force, hyperextension, and hyperflexion [20, 50]. Different injury mechanisms may still interfere with the results. Fourth, the knee joint is a three-dimensional structure. This study was conducted based on imaging data to analyze two-dimensional morphological indicators for PCL injury risk factors, which was limited by the measurement means and did not analyze three-dimensional morphological indicators. Fifth, 11 patients with patellar dislocation were compared with the remaining 81 patients in the PCL injury group, and this comparison may be unbalanced. In subsequent studies, we will add more samples to make corresponding comparisons. Last, no rigorous biomechanical experiments are performed to demonstrate the injury mechanism. Therefore, further large-scale multicenter studies are needed, and the trial design should be improved to clarify the risk factors for PCL injuries.

## Conclusion

Patients with PCL injuries had a larger patellar tilt angle, a lower intercondylar notch angle, and a longer posterior femoral condyle than patients with PCL integrity. The larger patellar tilt angle and lower intercondylar notch angle might be risk factors for PCL injuries.

## Data Availability

The datasets used and analysed during the current study are available from the corresponding author on reasonable request.

## Abbreviations

PCL: Posterior cruciate ligament
HKA: Hip-knee-ankle angle
NWI: Notch width index
PD: Patellar dislocation
ACL: Anterior cruciate ligament
MRI: Magnetic resonance imaging
CT: Computed tomography
ICC: Intraclass correlation coefficient
MFCR: Medial femoral condyle ratio
lFCR: Lateral femoral condyle ratio
